# Social Value Induction and Cooperation in the Centipede Game

**DOI:** 10.1371/journal.pone.0152352

**Published:** 2016-03-24

**Authors:** Briony D. Pulford, Eva M. Krockow, Andrew M. Colman, Catherine L. Lawrence

**Affiliations:** 1 Department of Neuroscience, Psychology and Behaviour, University of Leicester, Leicester, United Kingdom; 2 School of Psychology, Bangor University, Bangor, Wales, United Kingdom; Mälardalen University, SWEDEN

## Abstract

The Centipede game provides a dynamic model of cooperation and competition in repeated dyadic interactions. Two experiments investigated psychological factors driving cooperation in 20 rounds of a Centipede game with significant monetary incentives and anonymous and random re-pairing of players after every round. The main purpose of the research was to determine whether the pattern of strategic choices observed when no specific social value orientation is experimentally induced—the standard condition in all previous investigations of behavior in the Centipede and most other experimental games—is essentially individualistic, the orthodox game-theoretic assumption being that players are individualistically motivated in the absence of any specific motivational induction. Participants in whom no specific state social value orientation was induced exhibited moderately non-cooperative play that differed significantly from the pattern found when an individualistic orientation was induced. In both experiments, the neutral treatment condition, in which no orientation was induced, elicited competitive behavior resembling behavior in the condition in which a competitive orientation was explicitly induced. Trait social value orientation, measured with a questionnaire, influenced cooperation differently depending on the experimentally induced state social value orientation. Cooperative trait social value orientation was a significant predictor of cooperation and, to a lesser degree, experimentally induced competitive orientation was a significant predictor of non-cooperation. The experimental results imply that the standard assumption of individualistic motivation in experimental games may not be valid, and that the results of such investigations need to take into account the possibility that players are competitively motivated.

## Introduction

Cooperation and competition are the most quintessentially social forms of human behavior; in fact, it is difficult to think of any significant class of social interactions that does not involve cooperation or competition in some form. The Centipede game is an ideal tool for studying the motivational bases of reciprocal cooperation, because it is a dynamic game that presents players with greater scope for expressing cooperative/competitive, selfish/altruistic, trusting/distrustful, and individualistic/collective motives than other dyadic games, including the better known and more thoroughly researched Prisoner’s Dilemma game. The Prisoner’s Dilemma game also models some of these phenomena, but it is a simultaneous-choice game, whereas the Centipede game provides a dynamic model of alternating, cooperative interactions with potential longer-term benefits to both players.

**Fig 1:**
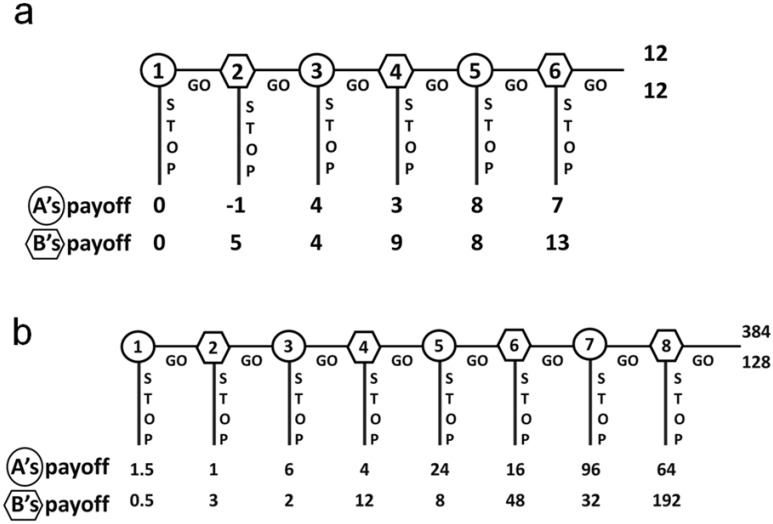
Typical Centipede games. (a) Linear Centipede game; (b) Exponential Centipede game. Decision nodes are numbered in circles for Player A and in hexagons for Player B.

The Centipede game provides a model of everyday relationships involving dynamic sequences of reciprocal cooperation between people who have full knowledge of each other’s actions. Opportunities for such reciprocity arise frequently in everyday life, as when academics take turns providing feedback on each other’s manuscripts and grant applications. The game was introduced by Rosenthal [1] and named by Binmore [2] after the resemblance of its game tree to a creature with many legs (see Fig 1). It is a dynamic game in which players move sequentially rather than simultaneously, in full knowledge of all previous moves that have been made. It provides a suitable experimental game for studying any social relationship in which one person (A) provides another (B) with a benefit at some cost to A, then B has the opportunity of reciprocating by providing A with a benefit at some cost to B, and so on for a finite number of moves, with all moves known to both players. The Prisoner’s Dilemma game cannot model such relationships, because it is a static game in which each player has only one move, the players move simultaneously rather than sequentially, and each player has to choose a move without knowing what the other player has chosen—features that are absent from the relationships of reciprocal cooperation that we are considering in this article.

An unarticulated default assumption in research using experimental games is that the preferences of the players match the payoff structures of the games that they are supposed to be playing, in other words, that the payoffs reflect their preferences. This is the case only if the players are individualistically motivated—motivated solely to maximize their own payoffs, without regard to their co-players’ payoffs. It is an essential assumption, because without it the choices of experimental participants are difficult to interpret according to the strategic properties of the experimental games under investigation, and consequently the investigators are unsure what games are actually being played. Recent research suggests that the assumption cannot be true in general, even when substantial monetary incentives are associated with the payoffs. Evidence is accumulating that players are often motivated by *other-regarding preferences* that take account of the payoffs to their co-players as well as their own. For example, it is now widely acknowledged that considerations of fairness and reciprocity frequently influence players’ strategy choices (e.g.,[3,4,5,6,7]). The aim of this article is to investigate individualistic and social motivations in the Centipede game, in particular to determine whether players in whom no social value orientation is specifically induced are motivated to maximize their individual payoffs, without other-regarding considerations, as assumed by default in game theory.

Fig 1(a) depicts the game tree of a Centipede game—actually a hexapod, but its length could be extended indefinitely without affecting its basic strategic properties. The game tree shows a sequence of *decision nodes*, represented by numbered circles and hexagons, starting at the left. Emerging from each decision node are two lines, labelled STOP and GO, representing the options facing the player whose turn it is to move. Players A and B alternate in choosing, at each decision node, whether to *defect* by choosing STOP or to *cooperate* by choosing GO. Whenever a player chooses the non-cooperative strategy and defects, the game immediately comes to an end, and the resulting payoffs are shown in the terminal nodes at the foot of the game tree below the corresponding decision nodes. If Player A defects by choosing STOP on the first move, then the game ends there, with both players receiving zero payoffs, as shown in the terminal node at the bottom of the centipede’s back leg. These payoffs may represent costs and benefits of any kind, but it is convenient to think of them as dollars, pounds sterling, euros, or other monetary units.

The particular version shown in Fig 1(a) is a *linear* Centipede game, because it has linearly increasing payoff sums: whenever a player cooperates by choosing GO, that player’s payoff is reduced by 1 unit, the co-player’s payoff increases by 5 units, and hence the size of the pot (the sum of both players’ payoffs at that node) increases by four units. Thus, if Player A cooperates by choosing GO at the first decision node, losing 1 unit of payoff and increasing Player B’s payoff by 5, and if Player B promptly defects by choosing STOP, then the game ends at that point, and the payoffs are –1 unit to Player A and 5 units to Player B, as shown at the bottom of the second leg. If both players cooperate at every decision node, then the game comes to a natural end after the sixth and final decision, with payoffs of 12 to each player.

The game shown in Fig 1(a) provides a model of alternating decision making in situations affording repeated opportunities for cooperation, a cooperative choice always imposing a small cost on the cooperator and providing a larger benefit to the recipient. A typical example in everyday life might be two neighbors who take turns looking after each other’s pets while the other is away on holiday. If the cost and benefit are both constant (1 and 5 respectively in this example), then the size of the pot increases linearly, as shown in the game tree, because the payoffs represent the *accumulating* costs and benefits. For example, if A looks after B’s pets while B takes a holiday, providing a benefit of 5 units to B at a cost of 1 unit to A, and if B then returns the favor, providing a benefit of 5 units to A at a cost of 1 unit to B, and if at that point A defects and terminates the relationship by leaving the area, for example, then the accumulated payoffs are shown in the third terminal node: 4 to A and 4 to B. The cost and benefits remain fixed, with payoffs accumulating as long as the relationship of reciprocal cooperation persists, and it is a merely technical aspect of the game that they are paid out only when one member of the dyad defects. Most people are very familiar with fluctuating bank accounts or investments, for example, in which accumulating costs and benefits are not crystallized until the cash is withdrawn or the stock is sold.

The principal variant is the *exponential* Centipede game, an example of which is shown in Fig 1(b). In this example, the value of the pot doubles whenever a player makes a cooperative GO move. This provides a model of reciprocal interactions involving sequential contributions to joint ventures that escalate in value. A typical example might be a relationship in which two software developers take turns working on a program that they intend to sell to a major company. If we assume that the program doubles in value with each additional contribution, and that the individuals involved have an agreement that allows either of them to sell the program, sharing the profit according to their accumulated contributions at any point, then we have the exponential Centipede game shown in Fig 1(b).

From a cursory examination of Fig 1(a) or 1(b), it is obvious that both players can earn large payoffs if both cooperate repeatedly. It is easy to see that the big money is on the right-hand side of the diagram, especially in Fig 1(b). Less obvious but nonetheless compelling is a logical argument, based on *backward induction*, apparently proving that a rational Player A should defect at the very first decision node, ending the game immediately with minimal payoffs to both players. This argument relies on the standard assumptions of game theory that both players are rational in the sense of being perfectly logical and always seeking to maximize their own payoffs, given their knowledge and beliefs at the time, and that this is *common knowledge* in the game, in the sense that both know it, both know that both know it, both know that both know that both know it, and so on, ad infinitum.

Suppose the game shown in Fig 1(b) has reached the final (eighth) decision node. A rational Player B will certainly defect, because the resulting payoff to Player B of 192 is a better payoff than 128 at the game’s natural end. According to the standard assumptions, Player A knows this. Consequently, at the previous decision node, Player A will defect in order to receive 96, rather than cooperate and receive 64 when Player B is certain to defect on the following move. When making a decision at the node before that, Player B knows all this and will therefore choose to defect, and so the argument unfolds back to the first decision node, proving that Player A will defect on the very first move of the game, even in a Centipede with 100 feet and enormous payoffs on its antennae protruding to the right. Defecting at the first decision node leads to the unique *subgame-perfect* (because it is arrived at by backward induction) *Nash equilibrium* (because it is an outcome in which neither player could have done better, given the co-player’s strategy) of the Centipede game.

This is a counterintuitive conclusion, but the backward induction argument has been authoritatively certified as logically sound [8,9]. According to the argument, a rational player will never cooperate, but will defect at the first available opportunity. A computer programmed to maximize its own payoffs with information that the co-player will do the same would presumably follow the logic of backward induction, given the assumptions of the argument. But the self-evident paradox of the game-theoretic solution suggests that human players are likely to approach the problem differently, as indeed they do.

Early experiments quickly established that most human players behave far more cooperatively than strictly rational players using backward induction [10,11,12,13], and this finding has been replicated in subsequent investigations (e.g., [14]). Only a very small minority of players defect on the first move (in the first experimental investigation of 6-node Centipede games, only 0.7% of all players exited at Node 1 [12]), and a substantial minority cooperate all the way to the end (in another early study [10], 14.5% of players reached the game’s natural end). Even chess grandmasters, who are familiar with backward induction from analyzing endgames, show cooperative behavior, according to a carefully conducted study [15]. There are evidently other, more human ways of approaching such problems than backward induction [16].

The Centipede game presents a paradox of cooperation; by definition, rational players always act to maximize their own individual payoffs, given their knowledge and beliefs, yet here we have a game in which irrational players who cooperate earn higher payoffs than rational players who defect. That is one of the reasons why the game provides an interesting vehicle for empirical research.

The assumption that decision makers are invariably motivated to maximize their individual payoffs is so deeply embedded that investigators sometimes take it for granted without even being aware of it. According to Fehr and Schmidt [6]: “Almost *all* economic models assume that all people are *exclusively* pursuing their material self-interest and do not care about ‘social’ goals per se” (p. 817, italics in original). However, the following examples show that it is not difficult to imagine circumstances in which even pure altruism, in the sense of maximizing the co-player’s payoffs at the expense of one’s own, can seem entirely natural. First, a doting grandparent playing a board game with a child may actively try to avoid winning in order to maximize the child’s payoffs in the game. Second, a jazz-loving man married to a classic music lover, choosing a recording as a present for his wife, in full knowledge that he will end up spending hours listening to whatever recording he buys, may decide to maximize his wife’s payoff by choosing a classical recording. Third, a woman who finds a child lost and crying in a shopping mall may take time to help find the child’s parent, even at the cost of being late for an appointment. In all of these examples, the first two borrowed from Colman et al. [17], it seems natural for players to strive to maximize their co-player’s payoffs rather than their own. The simplest interpretation in these examples is altruism, the most radical of other-regarding preferences, although other interpretations are always possible.

Von Neumann and Morgenstern [18] introduced into game theory an axiomatic concept of *expected utility* in which players’ payoff preferences are represented by numerical utilities, reflected by their actual choices. There can be no question but that players invariably strive to maximize their own expected utilities, because the theory defines their utilities according to the choices that they make; but utilities are not simply equivalent to selfish objective payoffs, because players may have preferences that are unselfish and other-regarding. In the examples discussed in the previous paragraph, for example, the utilities are evidently altruistic, and the actions taken reflect those preferences.

The psychological construct of *social value orientation* (SVO) was introduced by Messick and McClintock [19] and McClintock [20] to conceptualize both selfishness and other-regarding motivations. People’s orientations represent their preferences regarding the allocation of resources such as money between themselves and others, and also the motives that influence players’ strategy choices in games (for reviews, see [21,22]). The concept of SVO forms the foundation stone of *interdependence theory*, the only sustained attempt to provide a framework for understanding the full spectrum of selfish and other-regarding motivations of decision makers in games ([23,24,25]). Early research into SVO focused initially on decomposable two-player games and on individualistic, cooperative, and competitive orientations. Subsequently, altruism, equality-seeking, and other orientations were added to the theory, and a hybrid *prosocial* SVO, combining the cooperative and equality-seeking orientations, became a particular focus of attention (e.g., [26,27]).

In two-player games, SVO is generally defined in terms of payoff transformations, and this allows the following concise formalization [17]. Players’ utilities *U* are defined as functions of their own and their co-players’ objective payoffs *V*. If *v*_*i*_ and *v*_*j*_ are the objective payoffs to Players *i* and *j* in a two-player game, and *s*_*i*_ and *s*_*j*_ are strategies chosen by Players *i* and *j*, then Player *i* is assumed to maximize a real-valued utility function *U*_*i*_(*s*_*i*,_*s*_*j*_) = *f*_*i*_(*v*_*i*_,*v*_*j*_), and Player *i*’s SVO is a property of the particular function *f*_*i*_ that reflects the player’s motivation at the time. The *individualistic* SVO is represented by *f*_*i*_ = *v*_*i*_, the *altruistic* SVO by *f*_*i*_ = *v*_*j*_, the *cooperative* SVO by *f*_*i*_ = *v*_*i*_ + *v*_*j*_, the *competitive* SVO by *f*_*i*_ = *v*_*i*_−*v*_*j*_, and the *equality-seeking* SVO by *f*_*i*_ = min {*v*_*i*_−*v*_*j*_, *v*_*j*_−*v*_*i*_}. In the last case, either *v*_*i*_−*v*_*j*_ or *v*_*j*_−*v*_*i*_ must be negative if the two objective payoffs differ, and the equality-seeking player chooses a strategy that maximizes this negative difference, bringing it as close as possible to zero, which amounts to making the payoffs as equal as possible. According to this theoretical framework, players are invariably motivated to maximize their expected utilities, but these expected utilities may be individualistic, altruistic, cooperative, competitive, equality-seeking, or a combination of several different SVO utilities depending on the particular circumstances of the social interaction and individual differences between players.

In previous research, SVO has generally been interpreted as an individual difference variable. It has been found to correlate significantly with personality descriptions given by friends and roommates [28] and to predict activities in everyday life, such as volunteering for worthy causes [29,30]. Questionnaire measurements of trait SVO in several countries have found approximately 57% of people to be predominantly cooperative, 27% individualistic, and 16% competitive [31]. We shall call these individual differences *trait* SVO in contradistinction to *state* SVO that is induced by external circumstances, analogously to trait and state anxiety [32,33,34].

The earliest published experiment on social value orientations [19] was concerned with the effects of state and not trait SVO in decomposed experimental games. In this pioneering experiment, SVO was manipulated by a subtle difference in the instructions, where the co-player was referred to either as an “opponent” (to induce a competitive SVO) or a “partner” (to induce a cooperative SVO), and by displaying the players’ accumulated scores in different ways, calculated to draw attention to own payoffs, joint payoffs, or relative payoffs. Possible individual differences were explicitly ignored in that pioneering experiment. Shortly after its publication, McClintock [20] set out a theoretical basis for SVO in a system of propositions and corollaries that focused chiefly on state SVO: “The environment may operate to define the availability of outcomes to self and other in such a manner as to increase or decrease the likelihood that a given motivational predisposition will be dominant” (Proposition 5, p. 451). He discussed environmental “settings” that tend to evoke different orientations, including situations in which members of a family, team, organization, or nation feel under attack from outside and the cooperative SVO tends to be induced. He introduced the idea of individual differences only toward the end of the article, in his sixth and final proposition, where he considered it as an additional factor, over and above social contexts, that might help to explain social motives: “It seems indeed likely that some individuals are more predisposed to be individualistic, or cooperative, or competitive, or altruistic than others” (p. 453).

More recent researchers have manipulated state SVO in a variety of ways (e.g., [35,36,37,38,39]). Thus, although the majority of SVO research came to be focused on individual differences, and SVO is even sometimes defined as a stable preference (e.g., [40], p. 733), the experiments reported in this article investigated state rather than trait SVO. This may appear innovative, and it is indeed unusual in the context of resent research, but in fact our experiments only resurrect the original interpretation of SVO [19, 20]. The earlier notion of state SVO and its easy experimental manipulability raise the possibility of a direct test of the assumption that players in experimental games tend to default to the individualistic SVO, as often tacitly assumed in research using experimental games [41]. This simplifying assumption, commonly referred to as the *selfishness axiom* ([42], p. 797), is a defining property of rationality in neoclassical economics, orthodox decision theory, and game theory, although of course other interpretations of rationality are possible.

In the experiments described below, we manipulated state SVO with the aim of throwing light on what actually motivates players in repeated Centipede games in the absence of any specific state SVO induction. In Experiment 1, we induced cooperative and competitive orientations through framing experimental instructions and compared play in those conditions to play in a control condition without any specific state SVO induction. Each framing was expected to prime a particular SVO, because even subtle changes in the wording of task instructions have previously been found to prime specific mental mechanisms successfully in economic judgment and decision making [43]. Research further suggests that priming strongly affects behavior by influencing beliefs about other players’ likely strategies in the game [44]. In particular, a cooperative frame should lead participants to believe their co-players will choose cooperatively, thereby increasing trust and consequently also their own cooperation levels.

We hypothesized that an experimentally induced cooperative SVO would tend to increase cooperation because GO moves, incurring gains to the combined payoffs of both players, would invariably increase the utilities of team-oriented players. A competitive SVO, on the other hand, was predicted to decrease cooperation, because a player who tries to beat the other person by being the first to choose STOP would assign a higher risk to a cooperative GO move, given the possibility of game termination by the co-player at the following node. We made no specific prediction about behavior in the neutral treatment condition with no experimentally induced SVO, because the main purpose of the experiment is to discover how behavior in that condition would compare with behavior in the other conditions. In Experiment 2 we induced cooperative, competitive, and individualistic orientations, again with an additional control condition, and we also measured trait SVO. The individualistic SVO induction was expected to produce cooperation levels higher than the competitive condition and lower than the cooperative condition. Repeated cooperation increases both players’ payoffs, and if an individualistic player expects the co-player to reciprocate, then a GO move would be the obvious choice. Individualistic players might, in fact, mimic the strategy of cooperative players in order to elicit reciprocity and reach higher payoffs for themselves [12]. Hence, they are likely to cooperate for longer than competitive individuals. However, we expected lower cooperation levels compared to participants with cooperative SVO induction, because the risk of defection by the co-player increases toward the end and might eventually outweigh the temptation of higher payoffs. Furthermore, an individualistic player would have no incentive to cooperate at the final decision node, whereas very cooperative and altruistic individuals might choose GO in order to maximize the outcome of the team. Given the inherent asymmetry of the Centipede game, that at no point allows for a fair balancing of payoffs between the two players, we deliberately decided against inducing an equality-seeking SVO, and our analyses of trait SVO also omitted this type.

## Experiment 1

### Method

#### Participants

The participants were 60 students (45 women, 15 men) aged 18–39 years (*M* = 20.50, *SD* = 3.60) recruited partly from psychology undergraduates participating as a course requirement and partly through leaflets distributed across the university campus inviting volunteers to a “decision making experiment.” Ethical approval for the studies in the article was granted by the University of Leicester Psychology Research Ethics Committee and written consent was given by participants. Participants were remunerated according to a between-subjects version of the random lottery incentive system, a technique that has several advantages over other incentive schemes and has been shown to induce participants to play according to their payoff preferences [45,46,47,48]. After all testing sessions were completed, every participant was entered into a lottery in which three participants, selected at random, were paid the average payoff from all games that they played in the experiment, up to a theoretical maximum of £384.00 (US$620.00).

#### Design

Participants were assigned randomly to three treatment conditions involving different state SVO inductions—cooperative, competitive, and neutral—and were tested in groups of 10 in a computer-controlled experiment using custom software developed specifically for this study. Within each group, all participants received the same SVO induction. Each player pair played the game 20 times in succession (20 rounds of the game) with anonymous random re-pairing with another player in each round. The main dependent variable was the mean exit node, averaged across all 20 rounds. In each testing session, lasting between 20 and 40 minutes, half the players were randomly assigned the role of Player A and half to Player B for the whole session, and in their assigned roles they played 20 rounds of the exponential Centipede game shown in Fig 1(b). Rounds were divided into four blocks of five rounds each, and in each block, every Player A played exactly once against every Player B. To avoid carryover effects and reputation management tactics, this anonymous re-pairing of players was implemented differently in each trial block, and the participants did not know the identity of their co-player in any particular game (perfect stranger matching).

#### Materials and procedure

After signing consent forms, participants sat in front of individual computer monitors, and the game was displayed diagrammatically, as in Fig 1(b) but with decision nodes and payoffs displayed in different colors for Player A and Player B. Instructions, presented textually with examples, explained the rules of the game, the payoff functions, the number of rounds to be played, and the incentive scheme. Each participant was also given paper instructions displaying and explaining the Centipede game. For full materials see S3 File.

The wording of the instructions was identical across treatment conditions, apart from the following “reminder” inserted alongside instructions for making the first move in the game, repeated with instructions for the third move, and also repeated twice at the same locations in the paper instructions. In the competitive condition, the reminder drew attention to the players’ rivalry and introduced the notion of winning: “Please remember: Your decisions and those of the other person will determine who wins.” In the cooperative condition, the reminder drew attention to the payoffs to both players and introduced the notion of interdependence: “Please remember: Your decisions and those of the other person will determine how many points you both earn.” In the neutral condition, no reminder appeared at the corresponding positions in the on-screen or paper instructions.

The participants worked through the experiment at their own pace, apart from a constraint imposed by the anonymous re-pairing of players that required all games to be completed in any given round before the next round could begin. After completing 20 rounds against each of their co-players, participants were thanked, demographic information was collected, and email addresses were recorded. Once all testing sessions were complete, three participants were randomly selected and remunerated with the average of their payoffs across all of their games.

### Results

#### Exit nodes

**Fig 2:**
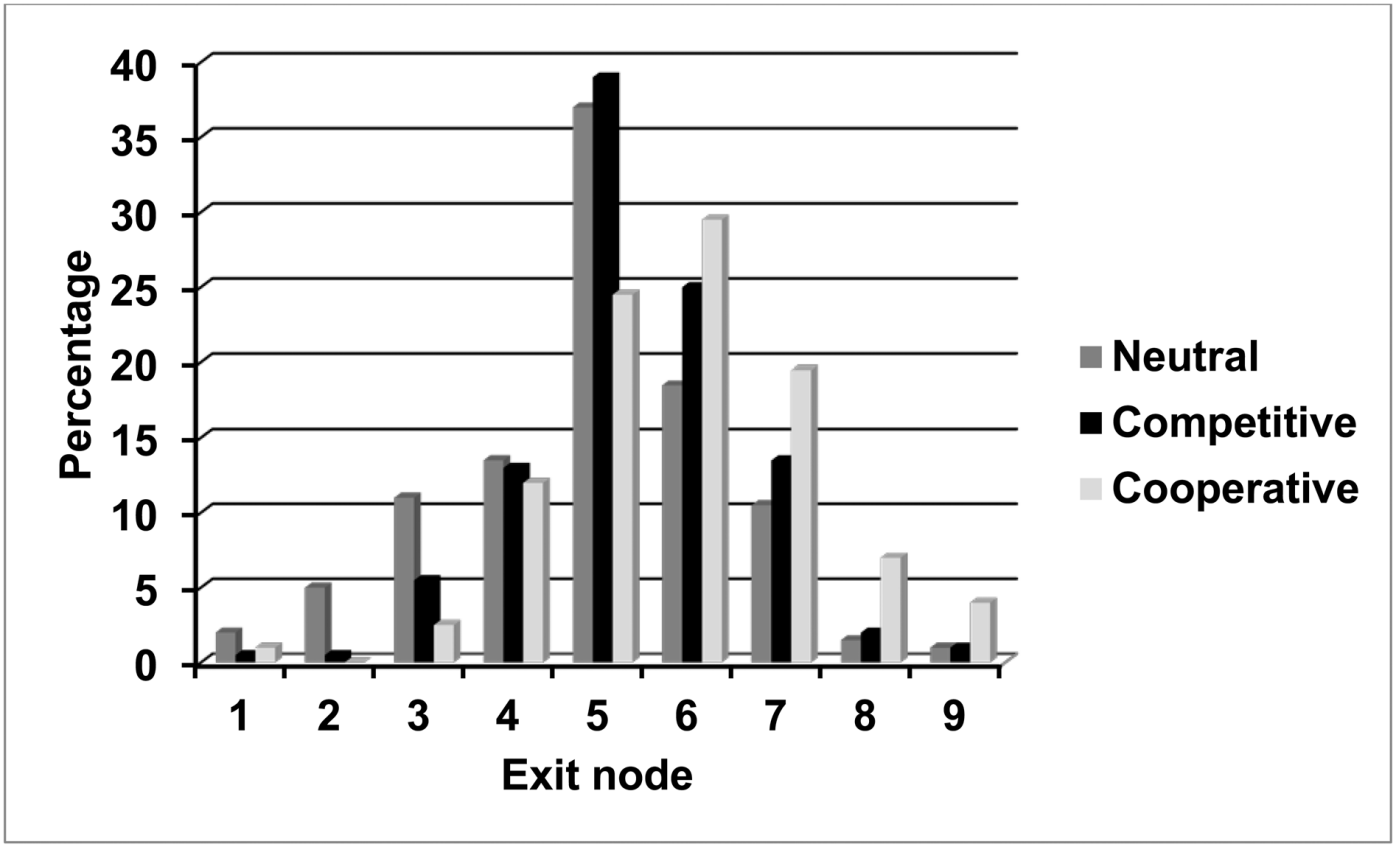
Exit Nodes: Experiment 1. Percentage of games terminating at each exit node in three treatment conditions of Experiment 1.

Raw data are available in the online Supporting Information (S1 and S2 Files). Fig 2 shows the percentage of games ending at each exit node, for games played in the three treatment conditions of the experiment. The game has eight decision nodes and nine exit nodes, the ninth arising if Player B chooses GO on the eighth decision node. The modal exit node was the fifth decision node in the neutral and competitive conditions and the sixth decision node in the cooperative condition. Very few games ended with a player exiting at the first decision node (1.17% of games played across all treatment conditions), and very few continued beyond the eighth decision node (2.00% across all treatment conditions). Non-cooperative play, shown by early exiting, was greatest in the neutral condition, and cooperative play, shown by late exiting, was greatest in the cooperative condition, with the competitive condition intermediate between the other two.

#### Effects of SVO induction

A one-way ANOVA examined the effect of treatment condition on mean exit node, taken from player pairs’ exit nodes. The mean exit node differed significantly across treatment conditions: *F*(2, 57) = 17.154, *p* < .001, partial η^2^ = .38. Tukey-HSD tests confirmed that all three conditions were significantly different from each other. The participants in the neutral condition (*M* = 4.90, *SD* = 0.44) exited significantly earlier (*p* = .02) than those in the competitive condition (*M* = 5.35, *SD* = 0.52), and both of these groups exited significantly earlier (*p* < .01) than those in the cooperative condition (*M* = 5.85, *SD* = 0.57).

#### Time series analysis

We measured cooperation over 20 rounds of the Centipede game, and there are possible differences between treatment conditions that are not captured by static analysis of means. Repeated measurements of a variate on successive occasions yields scores that are not independent but are potentially autocorrelated, with correlated error terms and non-stationary variances over repetitions. Standard statistical procedures assume independent and identically distributed data with zero autocorrelation, and they are consequently unsuitable for analyzing time series data. The appropriate analytic methods are specialized techniques of time series analysis (e.g., [49,50,51,52]).

**Fig 3:**
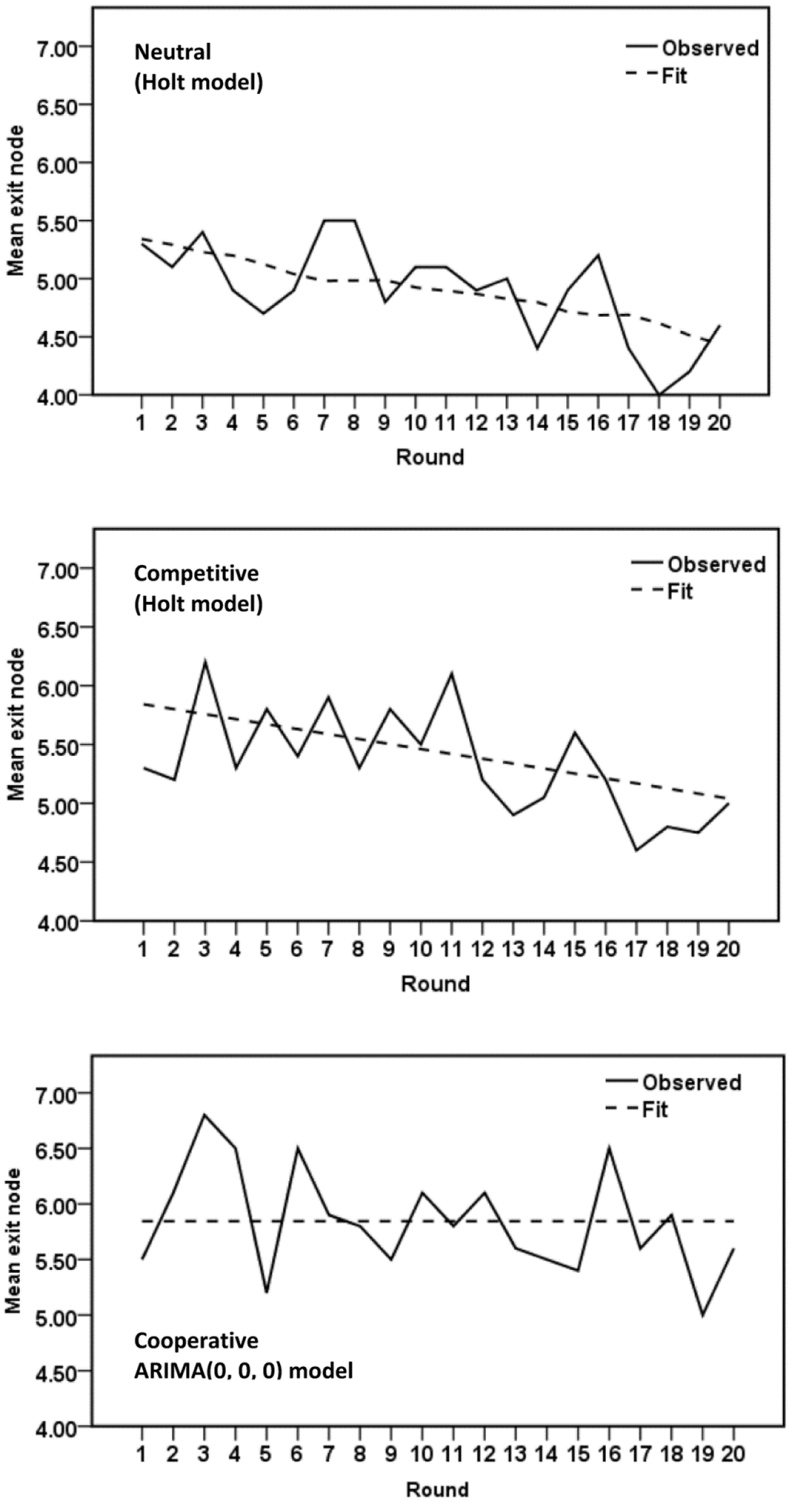
Time Series Analysis: Experiment 1. Time series sequence plots of mean exit nodes in Experiment 1.

To investigate the time course of cooperative moves within each treatment condition over the 20 rounds of the experiment, we performed time series analysis by fitting either an exponential smoothing model or an autoregressive integrated moving average (ARIMA) model—whichever provided the best fit—to the mean exit nodes, recorded for each round, in the neutral, competitive, and cooperative treatment conditions. The time series analysis was performed with the SPSS Expert Modeler. The Expert Modeler considers both exponential smoothing and ARIMA models. The goodness-of-fit measure is stationary *R*-square, a measure that compares the stationary part of the model to a simple mean model and is preferable to ordinary *R*-squared when there is a trend or other temporal structure in the time series. The observed time series and the best fitting models are shown graphically in Fig 3. The neutral and competitive conditions show downward-trending time series that appear very similar, apart from a slightly lower intercept in the neutral condition, and the cooperative condition shows no significant trend. For both the neutral and competitive treatment conditions, the Expert Modeler identified the exponential smoothing Holt model as the best fit. This particular exponential smoothing model is appropriate for series in which there is autocorrelation and linear trend. For the cooperative condition, in sharp contrast, the Expert Modeler identified an ARIMA(0, 0, 0) model, representing white noise without autocorrelation or trend, as the best fit. The absence of significant trend is indicated by the horizontal fit line in Fig 3.

### Discussion

The subgame-perfect Nash equilibrium, considered to be the unique rational solution of the Centipede game, was a poor predictor of the players’ choices in this experiment, as only a very small percentage of games ended at the first decision node. Choices in the cooperative condition were significantly more cooperative than those in the competitive condition, and choices in the competitive condition were significantly more cooperative than those in the neutral condition. This shows that cooperation was highly responsive to the very small nudges in the instructions that were used to induce state social value orientations in the cooperative and competitive treatment conditions. It also suggests that players who were given no specific motivational induction tended to adopt a generally non-cooperative pattern of play—more non-cooperative even than players in the competitive condition who were explicitly encouraged to beat the other player.

Why was play in the neutral condition so non-cooperative? Does it suggest that our SVO manipulation was not successful or that the framing of the task instructions was, in fact, not neutral? The effects in the treatment conditions in which we induced cooperative and competitive orientations yielded results more or less as expected. Participants in the neutral condition were free to play as cooperatively or non-cooperatively as they wished, and our results reveal that they tended to play comparatively non-cooperatively. One possibility is that participants, paired off and presented with sequences of decisions and substantial cash incentives, tended to interpret the task as a competitive game; furthermore, this may apply to experimental games in general. Participants in our neutral condition played significantly more non-cooperatively than those in the competitive SVO condition, although the difference was very small: 0.45 on an eight-point scale, or less than 6%. The instructions explicitly intended to induce competitiveness in the competitive SVO condition (“Please remember: Your decisions and those of the other person will determine who wins”) may have been milder than the interpretations that some of the participants in the neutral condition thought of for themselves, for example: “My decisions and those of the other person will determine whether I can beat my opponent into the ground.” Another point worth bearing in mind is that a competitive motivation does not necessarily or automatically lead to non-cooperative play and early defection, because players can increase the payoff difference in favor of themselves by delaying defection, provided the co-player does not defect first. Whatever the reason, it is an important finding that neutral instructions, characteristic of most experimental games, tended to induce comparatively non-cooperative play, at least in this case.

The time series analysis shows that the neutral condition, with no specific motivational induction, elicited a pattern of play over rounds of the game closely resembling play in the competitive condition, though slightly less cooperative. This seems inconsistent with a standard assumption in research using experimental games that, in the absence of any specific motivational induction, players default to the individualistic SVO. However, Experiment 1 did not include a specifically induced individualistic treatment condition. We therefore carried out a second experiment in which the individualistic state SVO was explicitly induced, to provide a deeper understanding of player motivations, and we also measured players’ trait SVO. In line with the standard assumption of behavioral game theory, we hypothesized that the neutral condition from Experiment 1 and the individualistic SVO condition from Experiment 2 would elicit similar behavior.

## Experiment 2

### Method

#### Participants

The participants were 114 students and employees at the University of Leicester (62 women, 52 men) aged 18–48 years (*M* = 22.61, *SD* = 4.86) recruited via an electronic bulletin board at the university. Participants were paid a £5.00 (US$8.00) show-up fee at the end of the testing session and were also incentivized with a between-subjects random lottery incentive, similar to the scheme used in Experiment 1. After all testing sessions were completed, three participants, selected at random, were remunerated with the average payoff from all games that they played in the experiment.

#### Design

Participants were assigned randomly to four treatment conditions, namely neutral, competitive, cooperative, and individualistic, and within each treatment condition were assigned randomly to the role of Player A or Player B for the whole testing session. Once again, the experiment was controlled by custom software developed for the purpose, and players were randomly and anonymously re-paired after each of the 20 rounds. The “reminders” inserted into the instructions in the cooperative and competitive conditions were identical to those used in Experiment 1. In the individualistic SVO condition, the reminder was: “Please remember: Your decisions and those of the other participant will determine how much money you receive for yourself.” The principal dependent variables were the mean exit node and the number of times (out of 20) that each participant defected by choosing STOP.

#### Materials and procedure

The experimental game was the same exponential Centipede game used in Experiment 1 (see Fig 1b), and the general procedure was the same. For full materials see S4 File. The on-screen instructions were presented in the form of an animated tutorial, with decision nodes and payoffs flashing when referred to in the textual instructions, and paper versions including a copy of the Centipede game diagram and a summary of the rules of the game, the payoff functions, the number of rounds to be played, and the incentive scheme. There were 28 participants in each of the cooperative, competitive, and individualistic conditions, and 30 in the neutral condition. The experiment was conducted over five one-hour testing sessions, with 16 to 32 participants per session. The participants sat at computer monitors and worked through the experiment at their own pace, except that, in each experimental round, to allow anonymous re-pairing of players, all games had to be completed before the next round could begin.

After the 20th round, participants were asked to complete the non-incentivized nine-item Triple-Dominance Measure of Social Values [40], one of the most commonly used measures of trait SVO. We used the instructions given in the appendix of [40], but we did not dichotomize the prosocial (cooperative and equality-seeking), individualistic, and competitive subscales of the Triple-Dominance Measure in the manner recommended by the scale developers, using six or more consistent responses out of nine as the criterion for each SVO type, because dichotomization is not considered to be best psychometric practice, partly because it involves sacrificing data [53]. Murphy and Ackerman [23] have specifically criticized the common practice of dichotomizing scores in the Triple-Dominance Measure. Instead, we followed the practice of carefully conducted recent studies (e.g., [54,55,56,57,58,59]) by simply using each player’s scores on the three subscales as the raw data.

On completion of the questionnaires, participants were paid their £5.00 show-up fees. At the end of all testing sessions, three participants were randomly selected and remunerated with their average payoff across all decision sequences.

### Results

#### Exit nodes

**Fig 4:**
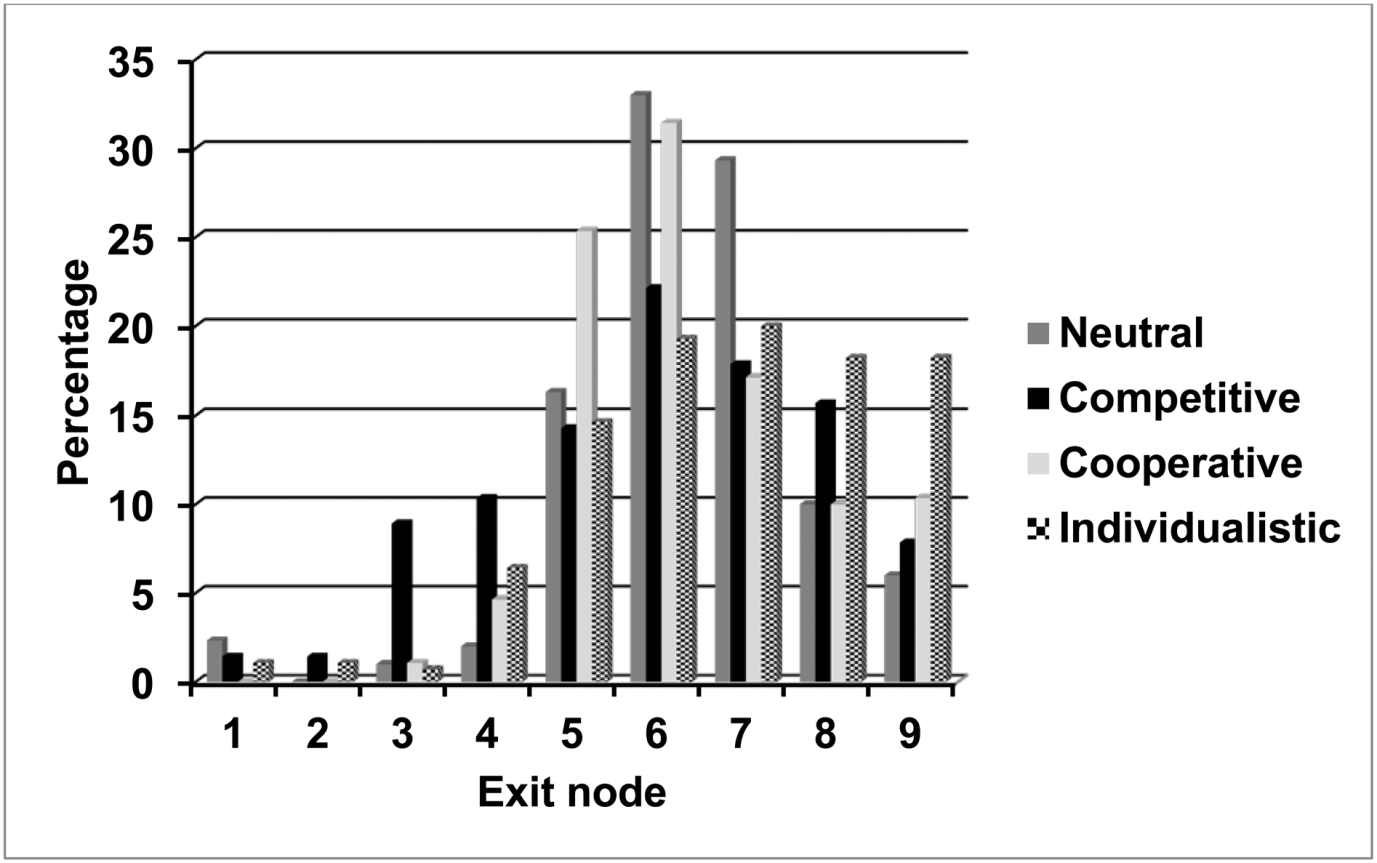
Exit Nodes: Experiment 2. Percentage of games terminating at each exit node in four treatment conditions of Experiment 2.

Raw data are available in the online Supporting Information. Fig 4 shows the percentage of games ending at each exit node for games played in the four treatment conditions of Experiment 2. Distributions in the neutral, competitive, and cooperative conditions were similar to Experiment 1 though slightly more cooperative. The sixth decision node of the game was the modal exit node in the cooperative condition (as in Experiment 1) and also in the neutral and competitive conditions, in which the fifth node was modal in Experiment 1. As in Experiment 1, very few games ended with a player exiting at the first decision node (1.23% of games played, across treatment conditions), but in all conditions, a larger percentage of games continued beyond the eighth decision node (10.53% across treatment conditions). Most striking are the data for the individualistic condition (absent from Experiment 1) in which more cooperative play occurred than in any other condition, including 18.21% of games continuing beyond the eighth decision node. Early exiting, indicative of non-cooperative play, was most frequent in the competitive condition, and late exiting, indicative of cooperative play, in the individualistic condition.

#### Effects of SVO induction

A one-way ANOVA examined the effect of treatment condition on mean exit node, taken from player pairs’ exit nodes. As in Experiment 1, the effect was significant, *F*(3, 110) = 2.676, *p* = .05, partial η^2^ = .07. The mean exit node was lower in the competitive condition (*M* = 5.98, *SD* = 0.99) than in the individualistic condition (*M* = 6.72, *SD* = 1.23), *p* = .028. Mean exit nodes in the cooperative condition (*M* = 6.30, *SD* = 0.95) and neutral condition (*M* = 6.32, *SD* = 0.68) were intermediate between the other two conditions. Post hoc Tukey-HSD tests confirmed that means for the cooperative and neutral conditions do not differ significantly from each other, or from the competitive or individualistic conditions (all *p* > .05); only the difference between the competitive and individualistic conditions differ significantly.

A forward stepwise multiple regression analysis revealed that trait and state SVO explained a significant proportion of the variance in mean exit nodes: *F*(3, 110) = 11.33, *p* < .001. Cooperative trait SVO was the most important (coefficient .77) predictor of cooperation, and competitive state SVO was a significant predictor (coefficient .15) of defection; no other trait or state SVO variable attained significance in the final model.

#### Time series analysis

**Fig 5:**
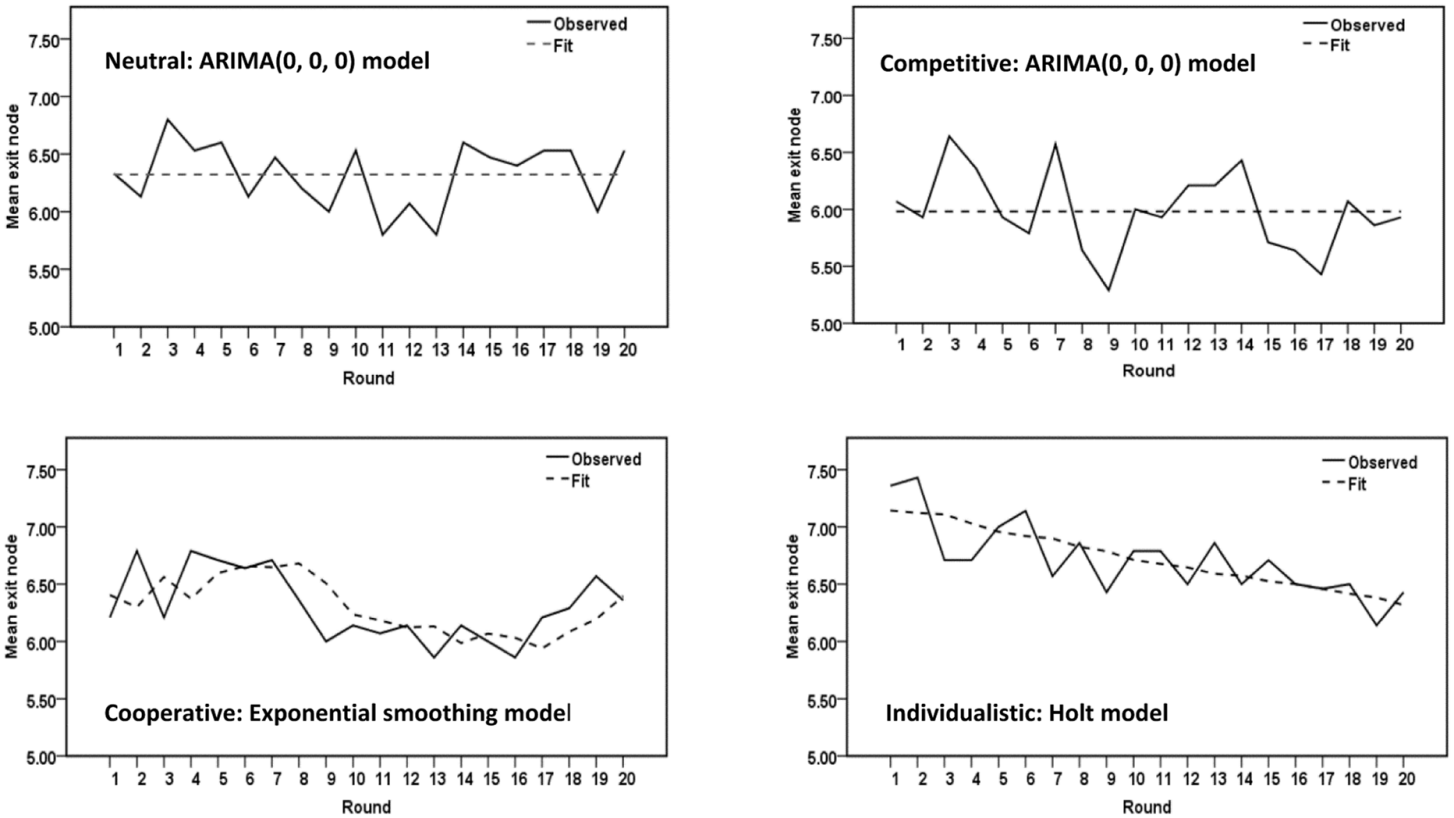
Time Series Analysis: Experiment 2. Time series sequence plots mean exit nodes in Experiment 2.

Time series sequence plots and best fitting models for Experiment 2 are shown in Fig 5. A visual examination of these plots suggest that the neutral and competitive treatment conditions generated similar patterns of strategy choices, with no significant trend over the 20 rounds of the experiment, although the mean level of cooperation was generally higher in the neutral than the competitive condition. The cooperative condition generated a significant curvilinear trend, and the individualistic condition a significant downward linear trend over rounds. Examining Figs 4 and 5, it is clear that although games played in the individualistic condition frequently continued to late exit nodes, the late exits occurred most frequently in early rounds, with games becoming steadily less cooperative over the 20 rounds of the experiment. However, the mean exit nodes remain higher even toward the end of the series than in almost any round in the competitive condition.

In the neutral and competitive conditions, the SPSS Expert Modeler identified ARIMA(0, 0, 0) as the best fit, suggesting the absence of any temporal structure in the data. The chief difference between these two time series is that the mean exit node was 6.32 in the neutral condition compared to 5.98 in the competitive condition. The results for these two treatment conditions suggest that there is no identifiable pattern in players’ strategy choices in either case, although the general level of cooperation is slightly higher in the neutral condition.

The cooperative condition generated the most complex time series, showing an unsteady increase in cooperation over the first few rounds, followed by a steep decrease, and then a sharp increase over the last few rounds. The best-fitting exponential smoothing model has one parameter, suggesting that scores on particular rounds were influenced by previous rounds, with considerable weighting on immediately preceding rounds.

The individualistic condition generated a time series that is best fit by an exponential smoothing Holt model, indicating a significant linear decline in cooperation, scores on each round being influenced by scores on previous rounds. Players began with higher levels of cooperation than in any of the other condition, and showed declining cooperation over the 20 rounds of the experiment, but remained relatively cooperative even towards the end.

#### Individual differences

We recorded the number of times out of a possible total of 20 that each player ended a game by defecting, and we then correlated these scores with the player’s scores on the Triple-Dominance Measure of trait SVO. For the computations that follow, the unit of analysis is the individual participant. There is no problem of interdependence between the number of defections of members of the same player pair, because only one player defected in each round, and players were randomly and anonymously re-paired after each round.

To disentangle the joint and separate effects of trait SVO scores on defection, we performed a standard multiple linear regression analysis, separately for each treatment condition, designating competitive, cooperative, and individualistic SVO questionnaire scores as predictor variables and number of defecting choices as the dependent variable. The significant predictor variables of the resulting four regression models are shown in Table 1, including unstandardized (*B*) and standardized (β) regression coefficients, multiple regression coefficients (*R*), and indices of shared variance (*R*^2^, and adjusted *R*^2^). In the neutral, cooperative, and individualistic treatment conditions, individualistic trait SVO score was a significant predictor of defection, higher scores tending to be associated with increased defection. In the competitive and individualistic conditions, competitive trait SVO score was a significant predictor of defection, higher scores once again tending to be associated with increased defection. Prosocial trait SVO score did not emerge as a significant predictor of defection in any of the four regression models.

**Table 1:** Multiple regression of number of defections over 20 rounds on competitive, cooperative, and individualistic SVO questionnaire scores in four treatment conditions.

Condition	Predictor	*B*	β	*t*	*p*	*R*	*R*^2^	Adj. *R*^2^
**Neutral**	Indiv.	.67	.53	3.28	.004	.55	.30	.25
**Competitive**	Comp.	.81	.40	2.22	.036	.47	.22	.16
**Cooperative**	Indiv.	.69	.63	3.97	.002	.62	.39	.34
**Individualistic**	Indiv.	.77	.46	2.66	.014	.54	.29	.24
	Comp.	.84	.39	2.27	.033			

### Discussion

As in Experiment 1, only a very small percentage of games ended at the first decision node prescribed by game theory. Once again, the state SVO inductions, minimal though they were, yielded significant differences between treatment conditions. Cooperative play was significantly higher in the individualistic condition than in the competitive condition.

Time-series analysis showed that the pattern of play over rounds was quite different in the individualistic condition than in any other of the treatment conditions. In the individualistic condition, choices were highly cooperative in early rounds, and this was followed by a linear decline in cooperation over rounds, but even in the final rounds choices were more cooperative than in any rounds in the competitive condition. In the neutral, competitive, and cooperative treatment conditions, patterns of play over rounds were all quite different, and the neutral condition, in particular, elicited a pattern resembling the competitive rather than the individualistic condition, without any downward trend in cooperation, though slightly more cooperative throughout.

Trait cooperative SVO and, to a lesser extent, state competitive SVO were both significant predictors of defection; people who are more cooperative by nature defected later, and being put in the competitive state lead to earlier defection. Multiple regression results suggest further that defection was influenced by individual differences in trait SVO in all four treatment conditions. In the neutral, cooperative, and individualistic conditions, players with highly individualistic trait SVO scores defected the most, and in the competitive and individualistic treatment conditions, highly competitive players defected the most. The fact that players with individualistic trait SVO defected the most may appear to contradict a finding reported earlier, that players in whom we induced individualistic state SVO cooperated the most. However, there is actually no contradiction, because it is possible to play cooperatively but nevertheless to defect frequently. In fact, the players who accumulated the highest individual payoffs were those who cooperate frequently, encouraging reciprocal cooperation from co-players, but also managed to be the first to defect in many games. Droste et al. [60] suggested that best-reply matching (matching the probability of playing a particular strategy to the probability that the strategy is a best reply) leads to highly cooperative play in Centipede games. Another practical playing policy to maximize individual payoff is to delay defection as late as one dares but to try to defect before the co-player.

One possible limitation worth noting is that the experimental state SVO induction could theoretically have influenced responses to the Triple-Dominance Measure of trait SVO, because the players completed the questionnaire after playing the Centipede game in one of the SVO induction conditions. We could not administer the questionnaire before the games were played, because the questionnaire items might have given the players ideas about how the payoffs could be interpreted, which in turn could have influenced their decisions, and it was their decisions that were the principal dependent variable under investigation. However, participants were assigned randomly to treatment conditions, and this enabled us to perform three ANOVAs to determine whether the cooperative, individualistic, and competitive trait SVO responses had, in fact, been influenced by the state SVO inductions, and all were non-significant (*p* > .05 in all three cases), leading us to have more confidence in our conclusions about the trait SVO measures.

## General Discussion and Conclusions

In both experiments, the cooperative state SVO induction led to significantly more cooperation, reflected in later mean exit nodes, than the competitive state SVO induction. This is to be expected, and it serves as a manipulation check, confirming that the SVO inductions elicited the intended motivational difference between these two groups, although they were implemented through small nudges inserted into the instructions. Drawing on previous research [43], these nudges may have influenced the players’ cooperative propensities either directly or indirectly, by altering their expectations of their co-players’ likely cooperativeness. In both experiments, the neutral treatment condition, in which no specific state SVO was induced, yielded results resembling the competitive condition.

Our most important finding is that the individualistic condition, introduced in Experiment 2, produced a pattern of choices strikingly different from the neutral condition, starting with extremely frequent cooperative choices on early rounds, followed by a decay in cooperation over rounds, though not sinking to the levels observed in the competitive condition throughout the 20 rounds of the game. This appears to refute the default assumption in most investigations using experimental games that, without any specific motivational induction, players can be assumed to be individualistically motivated. Indeed, in the absence of any specific SVO induction, they seem to resemble competitively motivated players more closely than individualistically motivated players, at least when playing the Centipede game. One possible interpretation is that individualistically motivated players cooperated on early rounds merely to keep the game going, with the underlying objective of maximizing their individual payoffs by defecting later, but before their co-players. Our findings are consistent with other evidence showing that players in Centipede games do not simply attempt to maximize their individual payoffs in the manner assumed by orthodox game theory [61].

In a forward stepwise multiple regression trait SVO was a significant predictor of cooperation, and trait SVO (with a smaller coefficient) was a significant predictor of non-cooperation. The results of regressing frequency of defections across 20 rounds of the game on players’ habitual competitiveness, cooperativeness, and individualism on a questionnaire measure of individual differences in trait SVO revealed that only individualism and competitiveness were significant predictors of defection. Individualism was predictive of defection in the neutral, cooperative, and individualistic treatment conditions, and competitiveness was predictive of defection in the competitive and individualistic conditions. This suggests, once again, that the motivational set of players in neutral treatment conditions, without any specific state SVO induction—the motivational set in many conventional experimental games—is psychologically and behaviorally distinct from the individualistic motivational set assumed by default in those experiments.

The choices of players differed slightly between Experiments 1 and 2 in the competitive, cooperative, and neutral conditions that were common to both experiments. In Experiment 1, the competitive SVO elicited the fewest cooperative choices, with a linear downward trend over rounds, compared to similarly infrequent cooperation but without any discernible downward trend in Experiment 2. In Experiment 1, the cooperative SVO elicited moderately cooperative play without any trend, compared to slightly more cooperation with a complicated curvilinear trend in Experiment 2. In Experiment 1, the neutral treatment condition elicited the least cooperation, declining linearly over rounds to extremely low levels, compared to intermediate cooperation without any trend over rounds in Experiment 2. How can these discrepancies be explained?

We believe that the discrepancies are probably attributable to differences in the way the experiments were administered. Experiment 1 was conducted in a modestly equipped laboratory, in small testing sessions (10 participants at a time), by a very young female experimenter, and many of the participants were fulfilling a course requirement through their participation. Experiment 2 was conducted in much larger testing sessions (16 to 32 participants at a time) by three much older experimenters, two female and one male, and none of the participants were fulfilling a course requirement. In Experiment 1, informal post-experimental feedback suggested that some of the participants were skeptical about the reality of the cash incentive on offer. Consequently, in Experiment 2, we went to some lengths, including showing the participants large wads of banknotes, to convince them that the incentives were real. Furthermore, Experiment 2 was conducted in a large well-equipped laboratory, with up-to-date computers and much more professional on-screen diagrams and instructions than Experiment 1. We believe that, as a consequence of all these differences, participants were probably more highly motivated and focused on the task in Experiment 2 than in Experiment 1, and that this difference explains the decay of cooperation in the neutral and competitive conditions in Experiment 1. If some participants in Experiment 1 become disengaged and keen to finish the task as quickly as possible, then early exiting may have been perceived as an obvious method of achieving this. It is worth commenting that a similar partial disengagement on the part of some participants is probably characteristic of many if not most psychological experiments.

The main conclusion to be drawn from the experiments reported in this article is that player motivation should not be taken for granted in experimental games. More specifically, we have established that the typical motivation of participants in the Centipede game, when no SVO is explicitly induced, is clearly not equivalent to the individualistic SVO that is implicitly assumed in almost all research on experimental games, and there is no obvious reason to believe that this finding applies only to the Centipede game. When no SVO is explicitly induced, players tend to be motivated rather non-cooperatively, whereas when an individualistic SVO is induced, they tend to begin very cooperatively and show learning in the form of declining cooperation over trials, converging slightly toward the subgame-perfect Nash equilibrium and spontaneously reacting to the hard lessons of seduction and betrayal as they pursue the twin objectives of eliciting as much cooperation as possible from their co-players and also being the first to defect. Whenever participants in experimental games are not individualistically motivated, they are not playing the games defined by the payoff functions presented to them. Behavioral game theorists and experimental investigators therefore need to be more circumspect than is customary in their assumptions about the motivations of their participants.

## Supporting Information

S1 File Data from Experiment 1. (XLSX)

S2 File Data from Experiment 2. (XLSX)

S3 File Supplemental Materials Experiment 1. (PDF)

S4 File Supplemental Materials Experiment 2. (PDF)
