# Correction: Social Value Induction and Cooperation in the Centipede Game

**DOI:** 10.1371/journal.pone.0155364

**Published:** 2016-05-05

**Authors:** Briony D. Pulford, Eva M. Krockow, Andrew M. Colman, Catherine L. Lawrence

There are errors in the funding section. The correct funding information is as follows: The research reported in this article was supported by the Leicester Judgment and Decision Making Endowment Fund http://www2.le.ac.uk/departments/psychology/research/JDMSP/leicester-judgment-and-decision-making-endowment-fund (Grant RM43G0176) to BDP and AMC. BDP is currently an administrator for this funding body. The funding agency had no role in study design, data collection and analysis, decision to publish, or preparation of the manuscript.
