# Supplementary material for: Social Value Induction and Cooperation in the Centipede Game
Source: PLoS One. 2016 Mar 24;11(3):e0152352. doi: 10.1371/journal.pone.0152352 (PMC4806875; doi:10.1371/journal.pone.0152352)
Supplement: S3 File — (PDF) [file pone.0152352.s003.pdf]

# Social value induction and cooperation in the Centipede game
## Supplemental Materials: Study 1

## Computer Instructions: Player 1, Competitive Frame

1.
"The experiment consists of four blocks, each made up of five individual decision making sequences. There are two different groups of players. During each of the blocks you will get paired up with each of the other group's members once. The order will be randomised and during the decision making sequences you will not know who the other player is.

Please press the space bar to continue."

2.
"Please have a look at the schematic display of the decision making sequence and the respective payoffs. There are eight different decision making nodes. Player 1 and Player 2 take turns making decisions, with Player 1 making the first move. They can decide between a go move and a stop move. Their decisions influence their respective monetary payoffs. If nobody stops the decision making sequence, it naturally finishes after the eighth decision node.
For the whole duration of the experiment, you will be Player 1.

Please press the space bar to continue."

3.
"Please remember that the payoffs represent real money amounts in pounds sterling. After completing the experiment, you have the chance to get awarded with the average amount of money you have earned in all the decision making sequences of the experiment. Therefore, please choose your moves carefully.

Also, please note that you are not allowed to talk to other participants for the duration of the experiment.

If you have further questions, please ask the experimenter now.

Please press the space bar to continue."

4.
"To indicate a "GO" move, please press the letter "H" on your key board.

To indicate a "STOP" move, please press the letter "J" on your key board.

Please press the space bar when you are ready to start your first decision sequence."

**Decision Sequences:**

1.
"Decision Sequence X
Please press the space bar to continue."

2.
"This is decision node number 1. Please make your move.

GO = "H"
STOP = "J"
Please remember: Your decisions and those of the other person will determine who wins."

3.
"This is decision node number 2. Please wait for Player 2 to make their move."

4.
"Player 2 chose "GO"."

5.
"This is decision node number 3. Please make your move.
GO = "H"
STOP = "J"
Please remember: Your decisions and those of the other person will determine who wins."

And so on until:

- Player 1 stops:
  "The decision sequence has now been stopped. Your payoff for this particular decision sequence is X. Please press the space bar and wait for the next decision sequence to start."
- Player 2 stops:
  "Player 2 chose "STOP". The decision sequence has now been stopped. Your payoff for this particular decision sequence is X. Please press the space bar and wait for the next decision sequence to start."
- Nobody stops:
  "You have now passed the eighth decision node. This is the end of the decision sequence. Your payoff is X. Please press the space bar and wait for the next decision sequence to start."

Respectively (after the completion of the last decision sequence):

- Player 1 stops:
  "The decision sequence has now been stopped. Your payoff for this particular decision sequence is X. Please press the space bar to continue."

- Player 2 stops:
  "Player 2 chose "STOP". The decision sequence has now been stopped. Your payoff for this particular decision sequence is X. Please press the space bar to continue."

- Nobody stops:
  "You have now passed the eighth decision node. This is the end of the decision sequence. Your payoff is X. Please press the space bar to continue."

After five decision sequences (one block):

"You have now completed the first/ second/ third block. Please press the space bar when you are ready to continue with the next block."

Respectively (after the completion of all four blocks):

"You have now completed the fourth and last block. Thank you very much for your participation. We will inform you via email if you are the winner of the financial reward. "

**Computer Instructions: Player 1, Cooperative Frame**

1.
“The experiment consists of four blocks, each made up of five individual decision making sequences. There are two different groups of players. During each of the blocks you will get paired up with each of the other group’s members once. The order will be randomised and during the decision making sequences you will not know who the other player is.

Please press the space bar to continue.”

2.
“Please have a look at the schematic display of the decision making sequence and the respective payoffs. There are eight different decision making nodes. Player 1 and Player 2 take turns making decisions, with Player 1 making the first move. They can decide between a go move and a stop move. Their decisions influence their respective monetary payoffs. If nobody stops the decision making sequence, it naturally finishes after the eighth decision node.
For the whole duration of the experiment, you will be Player 1.

Please press the space bar to continue.”

3.
“Please remember that the payoffs represent real money amounts in pounds sterling. After completing the experiment, you have the chance to get awarded with the average amount of money you have earned in all the decision making sequences of the experiment. Therefore, please choose your moves carefully.

Also, please note that you are not allowed to talk to other participants for the duration of the experiment.

If you have further questions, please ask the experimenter now.

Please press the space bar to continue.”

4.
“To indicate a “GO” move, please press the letter “H” on your key board.

To indicate a “STOP” move, please press the letter “J” on your key board.

Please press the space bar when you are ready to start your first decision sequence.”

**Decision Sequences:**

1.
“Decision Sequence X
Please press the space bar to continue.”

2.
“This is decision node number 1. Please make your move.
GO = “H”
STOP = “J”
Please remember: Your decisions and those of the other person will determine how many points you both earn.”

3.
"This is decision node number 2. Please wait for Player 2 to make their move."

4.
"Player 2 chose "GO"."

5.
"This is decision node number 3. Please make your move.
GO = "H"
STOP = "J"
Please remember: Your decisions and those of the other person will determine how many points you both earn."

And so on until:

- Player 1 stops:
  "The decision sequence has now been stopped. Your payoff for this particular decision sequence is X. Please press the space bar and wait for the next decision sequence to start."
- Player 2 stops:
  "Player 2 chose "STOP". The decision sequence has now been stopped. Your payoff for this particular decision sequence is X. Please press the space bar and wait for the next decision sequence to start."
- Nobody stops:
  "You have now passed the eighth decision node. This is the end of the decision sequence. Your payoff is X. Please press the space bar and wait for the next decision sequence to start."

Respectively (after the completion of the last decision sequence):

- Player 1 stops:
  "The decision sequence has now been stopped. Your payoff for this particular decision sequence is X. Please press the space bar to continue."
- Player 2 stops:
  "Player 2 chose "STOP". The decision sequence has now been stopped. Your payoff for this particular decision sequence is X. Please press the space bar to continue."
- Nobody stops:
  "You have now passed the eighth decision node. This is the end of the decision sequence. Your payoff is X. Please press the space bar to continue."

After five decision sequences (one block):

"You have now completed the first/ second/ third block. Please press the space bar when you are ready to continue with the next block."

Respectively (after the completion of all four blocks):

"You have now completed the fourth and last block. Thank you very much for your participation. We will inform you via email if you are the winner of the financial reward."

## Computer Instructions: Player 1, Neutral Frame

1.
"The experiment consists of four blocks, each made up of five individual decision making sequences. There are two different groups of players. During each of the blocks you will get paired up with each of the other group's members once. The order will be randomised and during the decision making sequences you will not know who the other player is.

Please press the space bar to continue."

2.
"Please have a look at the schematic display of the decision making sequence and the respective payoffs. There are eight different decision making nodes. Player 1 and Player 2 take turns making decisions, with Player 1 making the first move. They can decide between a go move and a stop move. Their decisions influence their respective monetary payoffs. If nobody stops the decision making sequence, it naturally finishes after the eighth decision node.
For the whole duration of the experiment, you will be Player 1.

Please press the space bar to continue."

3.
"Please remember that the payoffs represent real money amounts in pounds sterling. After completing the experiment, you have the chance to get awarded with the average amount of money you have earned in all the decision making sequences of the experiment. Therefore, please choose your moves carefully.

Also, please note that you are not allowed to talk to other participants for the duration of the experiment.

If you have further questions, please ask the experimenter now.

Please press the space bar to continue."

4.
"To indicate a "GO" move, please press the letter "H" on your key board.

To indicate a "STOP" move, please press the letter "J" on your key board.

Please press the space bar when you are ready to start your first decision sequence."

**Decision Sequences:**

1.
"Decision Sequence X
Please press the space bar to continue."

2.
"This is decision node number 1. Please make your move.
GO = "H"
STOP = "J""

3.
“This is decision node number 2. Please wait for Player 2 to make their move.”

4.
“Player 2 chose “GO”.”

5.
“This is decision node number 3. Please make your move.
GO = “H”
STOP = “J””

And so on until:

- Player 1 stops:
  “The decision sequence has now been stopped. Your payoff for this particular decision sequence is X. Please press the space bar and wait for the next decision sequence to start.”
- Player 2 stops:
  “Player 2 chose “STOP”. The decision sequence has now been stopped. Your payoff for this particular decision sequence is X. Please press the space bar and wait for the next decision sequence to start.”
- Nobody stops:
  “You have now passed the eighth decision node. This is the end of the decision sequence. Your payoff is X. Please press the space bar and wait for the next decision sequence to start.”

Respectively (after the completion of the last decision sequence):

- Player 1 stops:
  “The decision sequence has now been stopped. Your payoff for this particular decision sequence is X. Please press the space bar to continue.”
- Player 2 stops:
  “Player 2 chose “STOP”. The decision sequence has now been stopped. Your payoff for this particular decision sequence is X. Please press the space bar to continue.”
- Nobody stops:
  “You have now passed the eighth decision node. This is the end of the decision sequence. Your payoff is X. Please press the space bar to continue.”

After five decision sequences (one block):

“You have now completed the first/ second/ third block. Please press the space bar when you are ready to continue with the next block.”

Respectively (after the completion of all four blocks):

“You have now completed the fourth and last block. Thank you very much for your participation. We will inform you via email if you are the winner of the financial reward.”

Handout, Competitive

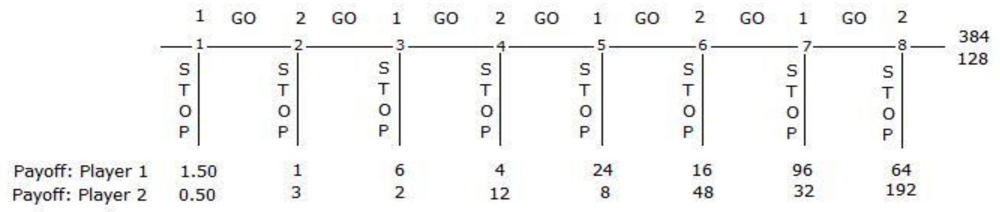


If you choose go at any point, you keep the decision sequence going.

If you choose stop at any point, you multiply your own payoff by six, you reduce the other person's payoff by a third, and you stop the decision sequence.

Your decisions and those of the other person will determine who wins.

Handout, Cooperative

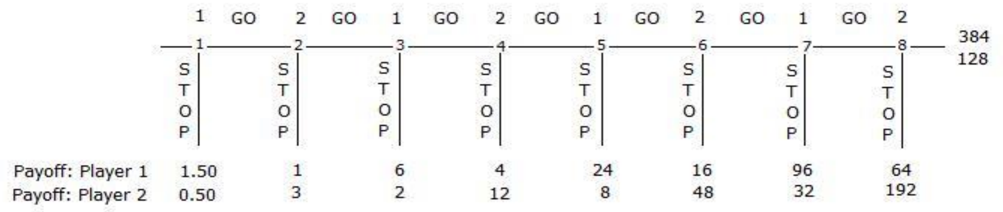


If you choose go at any point, you sacrifice one third from your own payoff, you multiply the other person's payoff by six, and you keep the decision sequence going.

If you choose stop at any point, you stop the decision sequence.

Your decisions and those of the other person will determine how many points you both earn.

Handout, Neutral

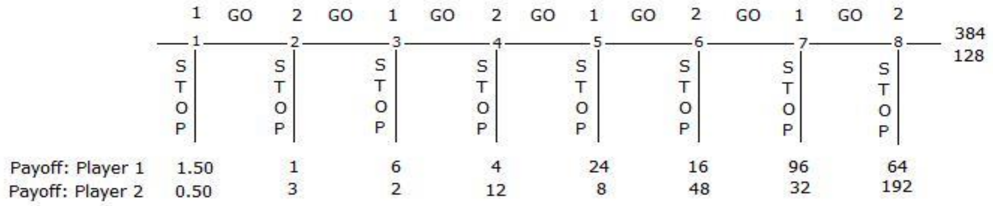


If you choose go at any point, you sacrifice one third from your own payoff, you multiply the other person's payoff by six, and you keep the decision sequence going.

If you choose stop at any point, you multiply your own payoff by six, you reduce the other person's payoff by a third, and you stop the decision sequence.
