# Supplementary material for: Social Value Induction and Cooperation in the Centipede Game
Source: PLoS One. 2016 Mar 24;11(3):e0152352. doi: 10.1371/journal.pone.0152352 (PMC4806875; doi:10.1371/journal.pone.0152352)
Supplement: S4 File — (PDF) [file pone.0152352.s004.pdf]

Social value induction and cooperation in the Centipede game
Supplemental Materials: Study 2

This is the 1st decision sequence.

[Condition 0 Neutral]

[Condition 1 Competitive] Please remember: Your decisions and those of the other participant will determine who wins the most money

[Condition 2 Cooperative] Please remember: Your decisions and those of the other participant will determine how much money you both receive

[Condition 3 individualistic] Please remember: Your decisions and those of the other participant will determine how much money you receive for yourself

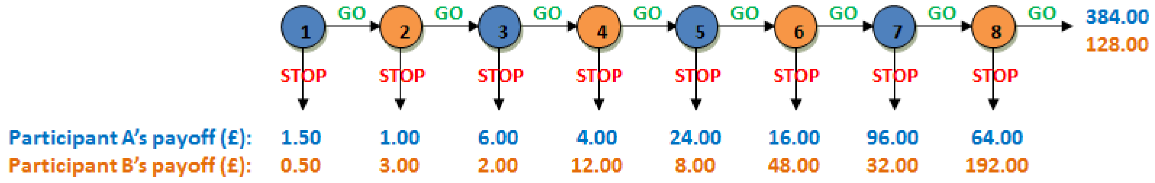

Participant A can choose to click on GO or STOP at 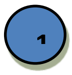

Participant A please make your decision now.

Participant B please wait for the outcome.

If Participant A chose to STOP, then:

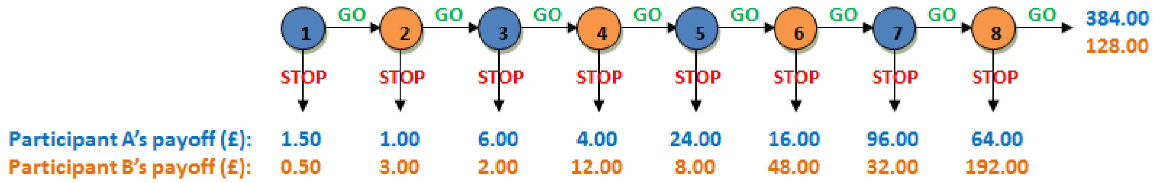

Participant A chose to **STOP** at 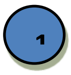

The decision sequence has ended.

Participant A's payoff is **£1.50**

Participant B's payoff is **£0.50**

You will now be randomly paired with a different participant and asked to make decisions to **GO** or **STOP** on the decision sequence again.

Randomly re-pair participants and begin new decision sequence.

If Participant A chose to GO, then:

Participant A chose to GO at 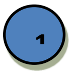

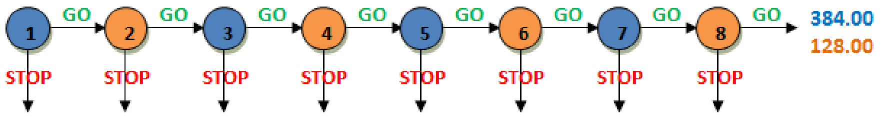

**Participant A's payoff (£):**	**1.50**	**1.00**	**6.00**	**4.00**	**24.00**	**16.00**	**96.00**	**64.00**
**Participant B's payoff (£):**	**0.50**	**3.00**	**2.00**	**12.00**	**8.00**	**48.00**	**32.00**	**192.00**

Participant B can choose to click on **GO** or **STOP** at 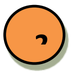

Participant B please make your decision now.

Participant A please wait for the outcome.

If Participant B chose to STOP, then:

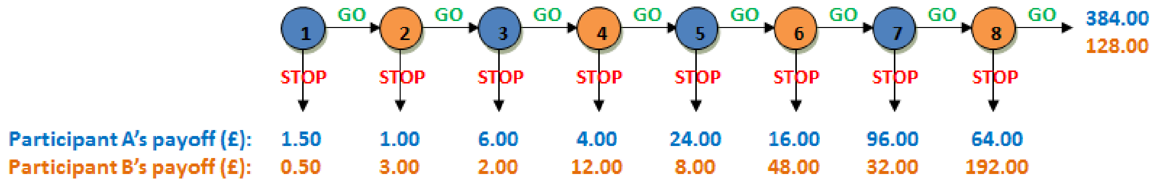

Participant B chose to **STOP** at 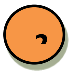

The decision sequence has ended.

Participant A's payoff is **£1.00**

Participant B's payoff is **£3.00**

You will now be randomly paired with a different participant and asked to make decisions to **GO** or **STOP** on the decision sequence again.

Randomly re-pair participants and begin new decision sequence.

If Participant B chose to GO, then:

Participant B chose to GO at 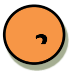

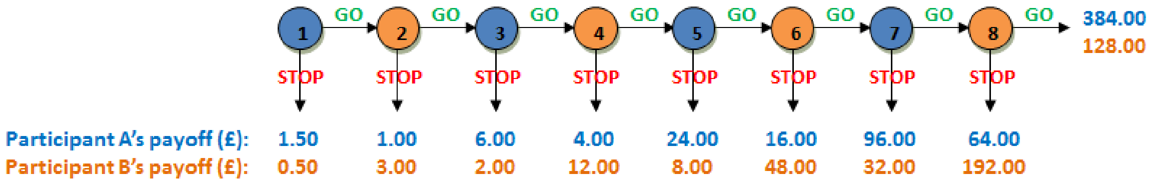

Participant A can choose to click on GO or STOP at 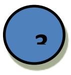

Participant A please make your decision now.

Participant B please wait for the outcome.

If Participant A chose to STOP, then:

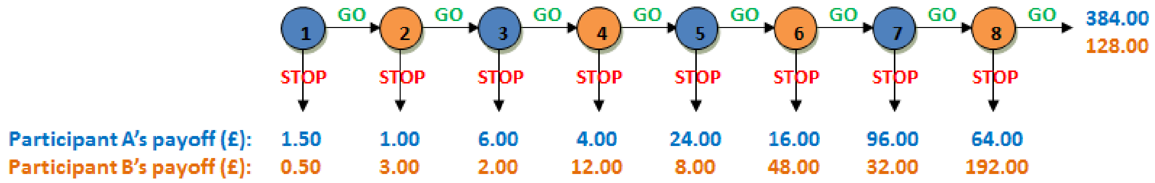

Participant A chose to **STOP** at 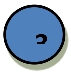

The decision sequence has ended.

Participant A's payoff is **£6.00**

Participant B's payoff is **£2.00**

You will now be randomly paired with a different participant and asked to make decisions to **GO** or **STOP** on the decision sequence again.

Randomly re-pair participants and begin new decision sequence.

If Participant A chose to GO, then:

Participant A chose to GO at 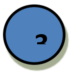

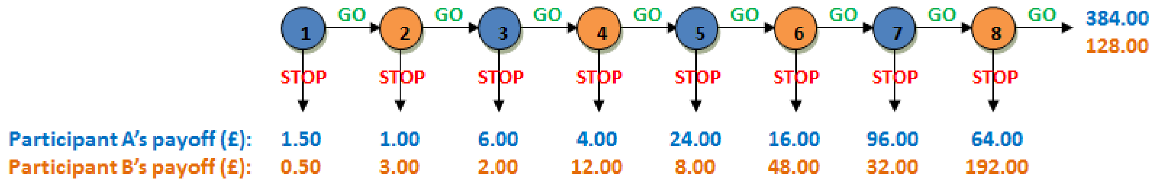

Participant B can choose to click on GO or STOP at 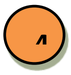

Participant B please make your decision now.

Participant A please wait for the outcome.

If Participant B chose to STOP, then:

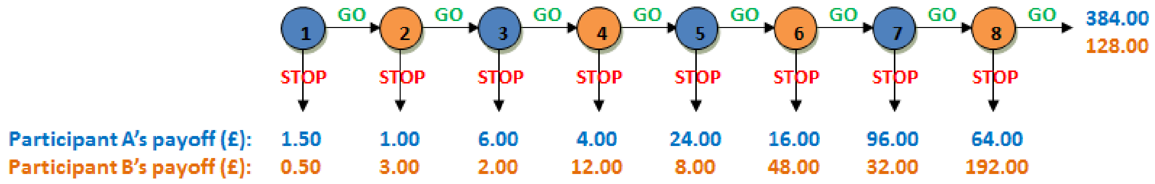

Participant B chose to **STOP** at 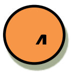

The decision sequence has ended.

Participant A's payoff is **£4.00**

Participant B's payoff is **£12.00**

You will now be randomly paired with a different participant and asked to make decisions to **GO** or **STOP** on the decision sequence again.

Randomly re-pair participants and begin new decision sequence.

If Participant B chose to GO, then:

Participant B chose to GO at 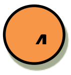

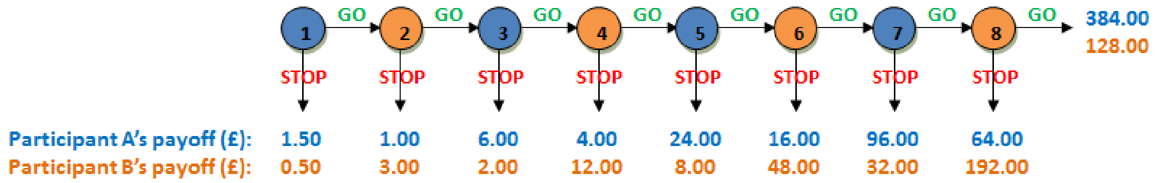

Participant A can choose to click on GO or STOP at 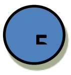

Participant A please make your decision now.

Participant B please wait for the outcome.

If Participant A chose to STOP, then:

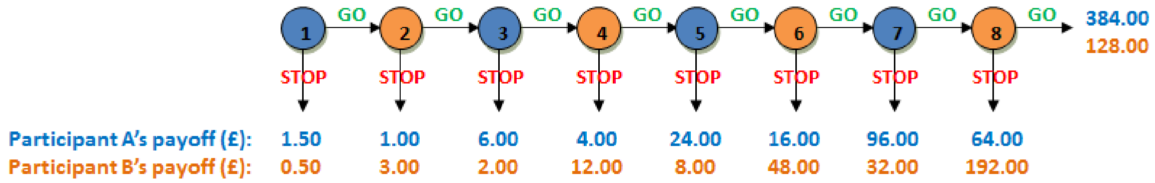

Participant A chose to STOP at 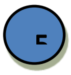

The decision sequence has ended.

Participant A's payoff is £24.00

Participant B's payoff is £8.00

You will now be randomly paired with a different participant and asked to make decisions to GO or STOP on the decision sequence again.

Randomly re-pair participants and begin new decision sequence.

If Participant A chose to GO, then:

Participant A chose to GO at 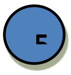

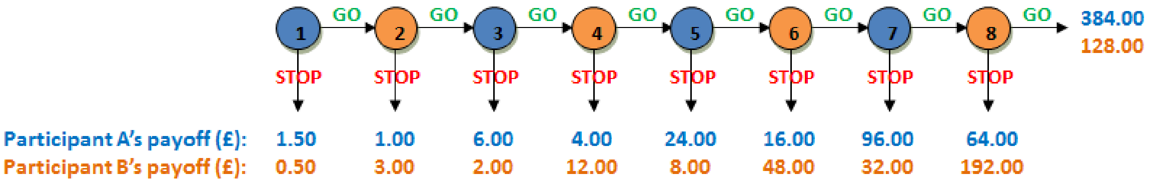

Participant B can choose to click on GO or STOP at 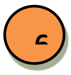

Participant B please make your decision now.

Participant A please wait for the outcome.

If Participant B chose to STOP, then:

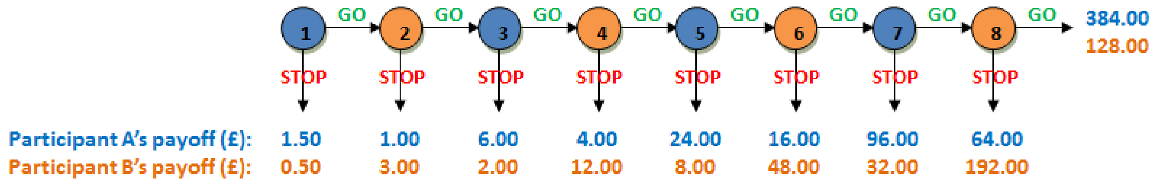

Participant B chose to **STOP** at 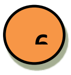

The decision sequence has ended.

Participant A's payoff is **£16.00**

Participant B's payoff is **£48.00**

You will now be randomly paired with a different participant and asked to make decisions to **GO** or **STOP** on the decision sequence again.

Randomly re-pair participants and begin new decision sequence.

If Participant B chose to GO, then:

Participant B chose to GO at 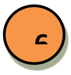

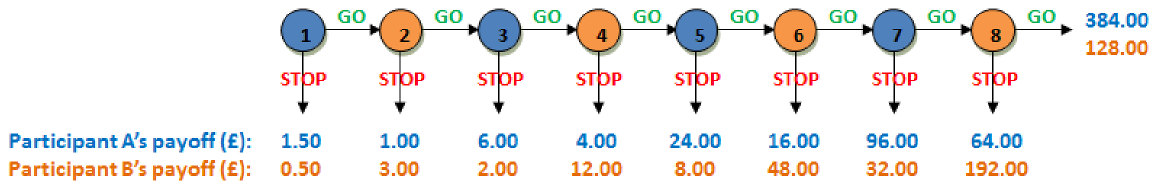

Participant A can choose to click on GO or STOP at 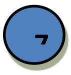

Participant A please make your decision now.

Participant B please wait for the outcome.

If Participant A chose to STOP, then:

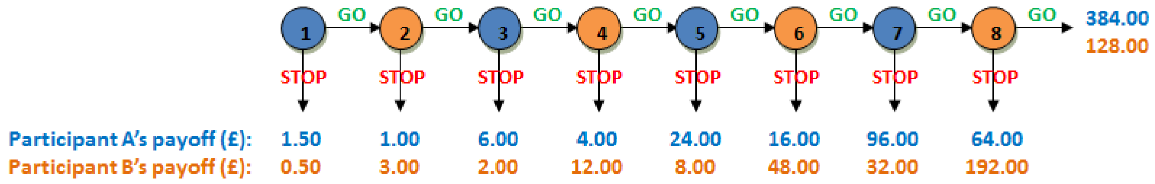

Participant A chose to **STOP** at 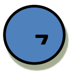

The decision sequence has ended.

Participant A's payoff is **£96.00**

Participant B's payoff is **£32.00**

You will now be randomly paired with a different participant and asked to make decisions to **GO** or **STOP** on the decision sequence again.

Randomly re-pair participants and begin new decision sequence.

If Participant A chose to GO, then:

Participant A chose to GO at 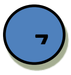

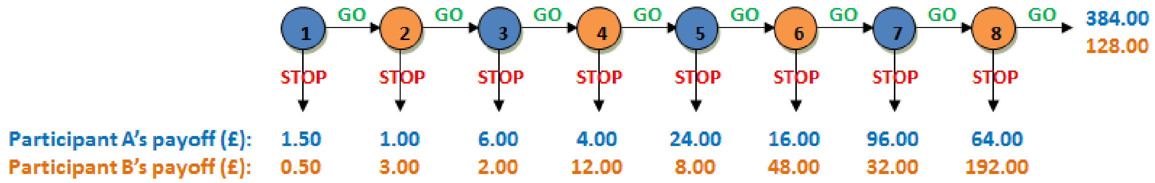

Participant B can choose to click on GO or STOP at 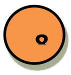

Participant B please make your decision now.

Participant A please wait for the outcome.

If Participant B chose to STOP, then:

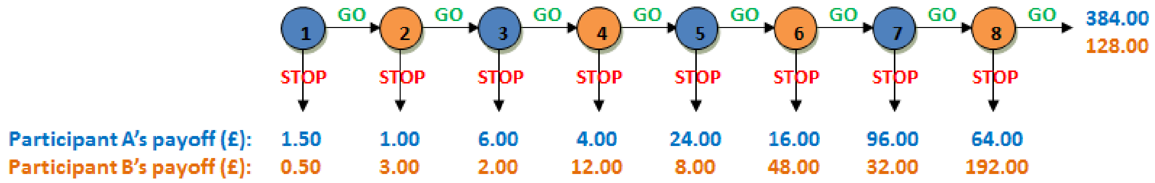

Participant B chose to **STOP** at 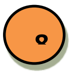

The decision sequence has ended.

Participant A's payoff is **£64.00**

Participant B's payoff is **£192.00**

You will now be randomly paired with a different participant and asked to make decisions to **GO** or **STOP** on the decision sequence again.

Randomly re-pair participants and begin new decision sequence.

If Participant B chose to GO, then:

Participant B chose to GO at 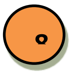

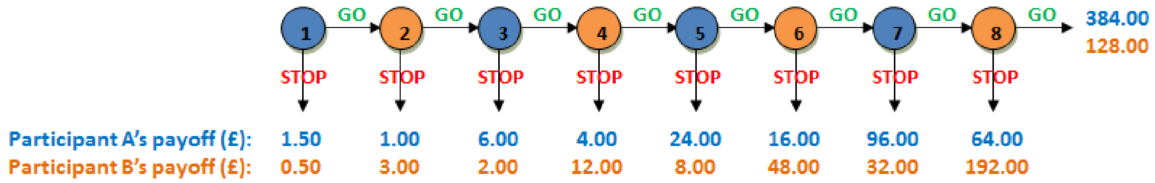

The decision sequence has naturally ended.

Participant A's payoff is **£384.00**

Participant B's payoff is **£128.00**

You will now be randomly paired with a different participant and asked to make decisions to **GO** or **STOP** on the decision sequence again.

Randomly re-pair participants and begin new decision sequence.

Before the start of each new decision sequence, participants are randomly re-paired.

This is the 2nd decision sequence.

[Condition 0]

[Condition 1] Please remember: Your decisions and those of the other participant will determine who wins the most money

[Condition 2] Please remember: Your decisions and those of the other participant will determine how much money you both receive

[Condition 3] Please remember: Your decisions and those of the other participant will determine how much money you receive for yourself

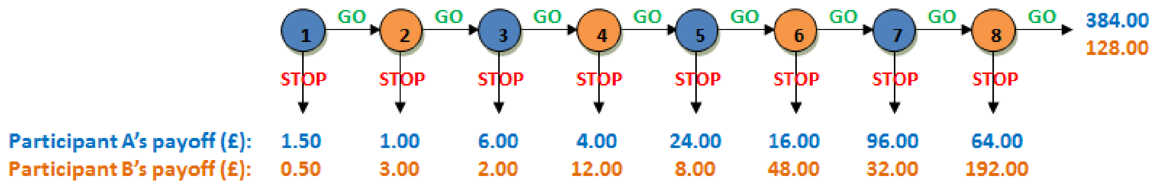

Participant A can choose to click on GO or STOP at 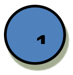

Participant A please make your decision now.

Participant B please wait for the outcome.

Etc up to:

This is the 20th decision sequence.

[Condition 0]

[Condition 1] Please remember: Your decisions and those of the other participant will determine who wins the most money

[Condition 2] Please remember: Your decisions and those of the other participant will determine how much money you both receive

[Condition 3] Please remember: Your decisions and those of the other participant will determine how much money you receive for yourself

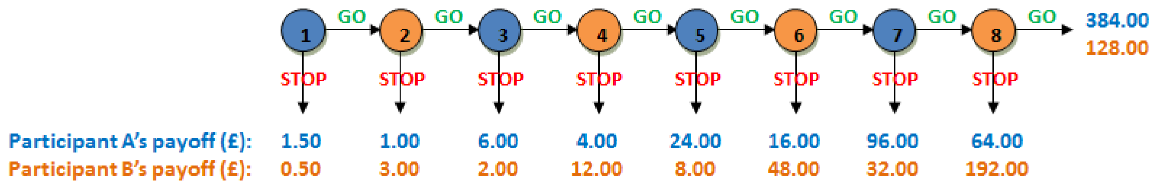

Participant A can choose to click on GO or STOP at 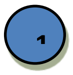

Participant A please make your decision now.

Participant B please wait for the outcome.

Final screen at the end of the 20th decision sequence.

You have now completed the final decision sequence.

Please click FINISH and complete the questionnaire that will be displayed on the screen.
